# Guias alimentares: estratégia para redução do consumo de alimentos ultraprocessados e prevenção da obesidade

**DOI:** 10.26633/RPSP.2019.59

**Published:** 2019-12-16

**Authors:** Gisele Ane Bortolini, Ana Luisa de Paiva Moura, Ana Maria Cavalcante de Lima, Helissa de Oliveira Mendonça Moreira, Olivia Medeiros, Isabel Cristina Moutinho Diefenthaler, Michele Lessa de Oliveira

**Affiliations:** 1 Ministério da Saúde Coordenação Geral de Alimentação e Nutrição BrasíliaDF Brasil Ministério da Saúde, Coordenação Geral de Alimentação e Nutrição, Brasília (DF), Brasil.

**Keywords:** Guias alimentares, política pública, promoção da saúde, obesidade, Brasil, Food guide, public policy, health promotion, obesity, Brazil, Guías alimentarias, política pública, promoción de la salud, obesidad, Brasil

## Abstract

A obesidade é um grave problema de saúde pública e seu aumento tem sido associado ao crescimento do consumo de ultraprocessados em diversas regiões, incluindo a América Latina. Os guias alimentares são diretrizes oficiais para a promoção da alimentação saudável e podem servir como ferramentas para a prevenção da obesidade. O Brasil foi o primeiro país a adotar o nível de processamento para classificar os alimentos e fazer recomendações a partir dessa classificação em seus guias alimentares. Assim, o objetivo deste artigo é compartilhar a experiência brasileira na incorporação de tais recomendações como diretrizes oficiais e na elaboração de guias alimentares. Para o desenvolvimento dos guias alimentares brasileiros, o Ministério da Saúde buscou valorizar a construção coletiva, com participação e discussão do conteúdo por atores estratégicos. No Guia Alimentar para Crianças Brasileiras Menores de 2 Anos houve aprimoramento do processo, com formação de um comitê gestor e um comitê de monitoramento político, chamada pública de pesquisadores e profissionais de saúde e oficinas de escuta com atores-chave para a definição do escopo do guia. O processo de validação contou com oficinas adicionais, com presença de atores estratégicos, além de oficinas em todos os estados para debate e mobilização para participação na consulta pública *on-line*. Como resultado do aprendizado, destacamos a importância da participação de atores estratégicos e a necessidade de dar ampla transparência ao processo de elaboração e validação de guias alimentares.

## Abstract (en)

Obesity is a serious public health problem whose increase has been associated with the growing consumption of ultra-processed foods in several world regions, including Latin America. Food guides are official guidelines used to promote healthy eating habits and may serve as a tool to prevent obesity. Brazil was the first country to adopt the level of processing to categorize food items and make recommendations based on these categories in its food guides. Thus, the objective of the present article is to share the Brazilian experience with incorporating processed food recommendations as official guidelines and with the development of food guides. In the development of Brazilian food guides, the Ministry of Health emphasized a collective construction, with participation and discussion of contents by strategic stakeholders. The process was refined in the Food Guide for Brazilian Children below 2 Years of Age, with establishment of a management committee and a political monitoring committee, public call for participation by researchers and health care professionals, and input workshops with key stakeholders to define scope. The validation process relied on additional input workshops with key stakeholders, in addition to workshops held in all Brazilian states for debate and mobilization for online public consultation. Highlights of this learning process included the involvement of key stakeholders and the importance of transparency across all steps of development and validation of food guides.

## RESUMEN

La obesidad es un grave problema de salud pública cuyo aumento se ha relacionado con el incremento del consumo de alimentos ultraprocesados en diversas regiones del mundo, incluida América Latina. Las guías alimentarias son directrices oficiales usadas para promover la alimentación saludable y pueden servir de herramienta para prevenir la obesidad. Brasil fue el primer país en adoptar el grado de procesamiento como criterio para clasificar los alimentos y formular recomendaciones en sus guías alimentarias usando como base esa clasificación. El objetivo de este artículo es compartir la experiencia brasileña al incorporar esas recomendaciones como directrices oficiales y elaborar las guías alimentarias. En la elaboración de las guías alimentarias brasileñas, el Ministerio de Salud valoró la construcción colectiva, a saber, la participación de actores estratégicos y el debate que estos actores entablaron sobre sus contenidos. En la *Guía alimentaria para los niños brasileños menores de dos años*, el proceso se perfeccionó gracias al establecimiento de un comité gestor y un comité de monitoreo político, un llamamiento público a la participación de investigadores y profesionales de salud, y la realización de talleres participativos con actores clave para definir el objetivo de la directriz. Para el proceso de validación se realizaron otros talleres, con la presencia de actores estratégicos, así como talleres realizados en todos los estados del país a fin de fomentar un debate y promover la participación en una consulta pública en línea. Como resultado de este aprendizaje, se destaca la importancia de que participen los actores estratégicos y la necesidad de que el proceso de elaboración y validación de las guías alimentarias sea transparente.

A obesidade é uma das principais preocupações em termos de saúde pública no mundo (1). O aumento da obesidade tem sido associado ao crescimento da venda e do consumo de alimentos ultraprocessados em diversas regiões, inclusive na América Latina (2).

O Brasil foi o primeiro país a adotar, em suas diretrizes oficiais, o nível de processamento dos alimentos como forma de fazer recomendações de alimentação saudável – como demonstram o Guia Alimentar para a População Brasileira (3) e, mais recentemente, o Guia Alimentar para Crianças Brasileiras Menores de 2 Anos (4). Além de servirem como instrumento para incentivar práticas alimentares saudáveis no âmbito individual e coletivo, esses guias são indutores de políticas públicas que visam a fomentar, apoiar e proteger a saúde e a segurança alimentar e nutricional da população brasileira.

Nesse contexto, o objetivo deste artigo é compartilhar a experiência brasileira relativa à adoção de recomendações de alimentação saudável com base no nível de processamento dos alimentos e relatar o processo de elaboração do Guia Alimentar para Crianças Brasileiras Menores de 2 Anos, que aprimorou a experiência de elaboração do Guia de 2014.

## CLASSIFICAÇÃO DOS ALIMENTOS COM BASE NO NÍVEL DE PROCESSAMENTO

Historicamente, as recomendações de alimentação e nutrição têm-se baseado na composição nutricional dos alimentos, enfatizando ora a importância do balanço de nutrientes, ora a predominância de determinados nutrientes na composição dos diferentes grupos de alimentos. No entanto, nos últimos anos, estudos que analisam a relação entre alimentos, nutrição, saúde e doença mostraram que a extensão e o propósito do processamento dos alimentos estão relacionados com a atual pandemia de obesidade e outras doenças crônicas, como câncer e síndrome do intestino irritável (5-9).

Considerando esses aspectos, o Ministério da Saúde no Brasil adotou uma classificação de alimentos baseada no seu nível de processamento. A classificação foi desenvolvida por um grupo de pesquisadores da Universidade de São Paulo (USP), que também contribuiu para a elaboração e a redação da segunda edição do Guia Alimentar para a População Brasileira (3).

Os princípios considerados na elaboração do Guia foram: alimentação é mais do que ingestão de nutrientes; recomendações de alimentação devem estar em sintonia com seu tempo; alimentação adequada e saudável deriva de um sistema alimentar social e ambientalmente sustentável; diferentes saberes geram conhecimento para a formulação de guias alimentares; e guias alimentares ampliam a autonomia nas escolhas alimentares. A regra de ouro da recomendação de alimentação saudável para a população do país, adotada no guia alimentar brasileiro, é a seguinte: prefira sempre alimentos *in natura* ou minimamente processados e preparações culinárias a alimentos ultraprocessados. A classificação dos alimentos conforme essa lógica aparece na tabela 1. No Guia Alimentar para Crianças Brasileiras Menores de 2 Anos, além dessas mensagens, ganhou destaque a mensagem central de que alimentos ultraprocessados não devem ser ofertados antes de 2 anos de idade (4).

**TABELA 1.** Classificação dos alimentos de acordo com o nível de processamento, Brasil, 2019

Grau de processamento/definição	Exemplos
Alimentos *in natura* ou minimamente processados	
Obtidos diretamente de plantas ou de animais. Não sofrem qualquer alteração após deixar a natureza, podendo ser submetidos apenas a processos como limpeza, remoção de partes não comestíveis ou indesejáveis, fracionamento, moagem, secagem, fermentação, pasteurização, refrigeração, congelamento e processos similares que não envolvam agregação de sal, açúcar, óleos, gorduras ou outras substâncias ao alimento original.	Hortaliças, frutas, raízes e tubérculos, cereais, leguminosas, carnes, ovos, leite e água
Óleos, gorduras, açúcar e sal	
Extraídos de alimentos *in natura* e geralmente não consumidos isoladamente, mas usados para temperar e cozinhar alimentos e para criar preparações culinárias.	Óleos vegetais, manteiga, banha de porco, açúcar e sal
Alimentos processados	
Alimentos *in natura* manipulados industrialmente com adição de sal ou açúcar ou outra substância de uso culinário que aumenta a durabilidade e torna esses alimentos mais agradáveis ao paladar	Vegetais preservados em salmoura ou solução de sal e vinagre Extrato ou concentrados de tomate com adição de sal ou açúcar Frutas em calda e cristalizadas Carne seca e toucinho, sardinha e atum enlatados Queijos Pães feitos de farinha de trigo, leveduras, água e sal
Alimentos ultraprocessados	
Formulações industriais feitas inteiramente ou majoritariamente com substâncias extraídas de alimentos (óleos, gorduras, açúcar, amido, proteínas), derivadas de constituintes de alimentos (gorduras hidrogenadas, amido modificado) ou sintetizadas em laboratório com base em matérias orgânicas como petróleo e carvão (corantes, aromatizantes, realçadores de sabor e vários tipos de aditivos usados para dotar os produtos de propriedades sensoriais atraentes). Envolvem, geralmente, técnicas de manufatura e aditivos de uso exclusivamente industrial.	Biscoitos, sorvetes, balas e guloseimas em geral Cereais açucarados Misturas para bolo Barras de cereal Macarrão e temperos instantâneos, salgadinhos de pacote Refrescos e refrigerantes, iogurtes e bebidas lácteas adoçados e aromatizados, bebidas energéticas Produtos congelados e prontos para aquecimento como pratos de massas, pizzas, hambúrgueres, empanados do tipo *nuggets*, salsichas e outros embutidos Pães e produtos panificados cujos ingredientes incluem substâncias como gordura vegetal hidrogenada, açúcar, amido, soro de leite, emulsificantes e outros aditivos

***Fonte***: Guia Alimentar para a População Brasileira (3).

## ETAPAS DO DESENVOLVIMENTO DO GUIA ALIMENTAR PARA CRIANÇAS

**FIGURA 1.**
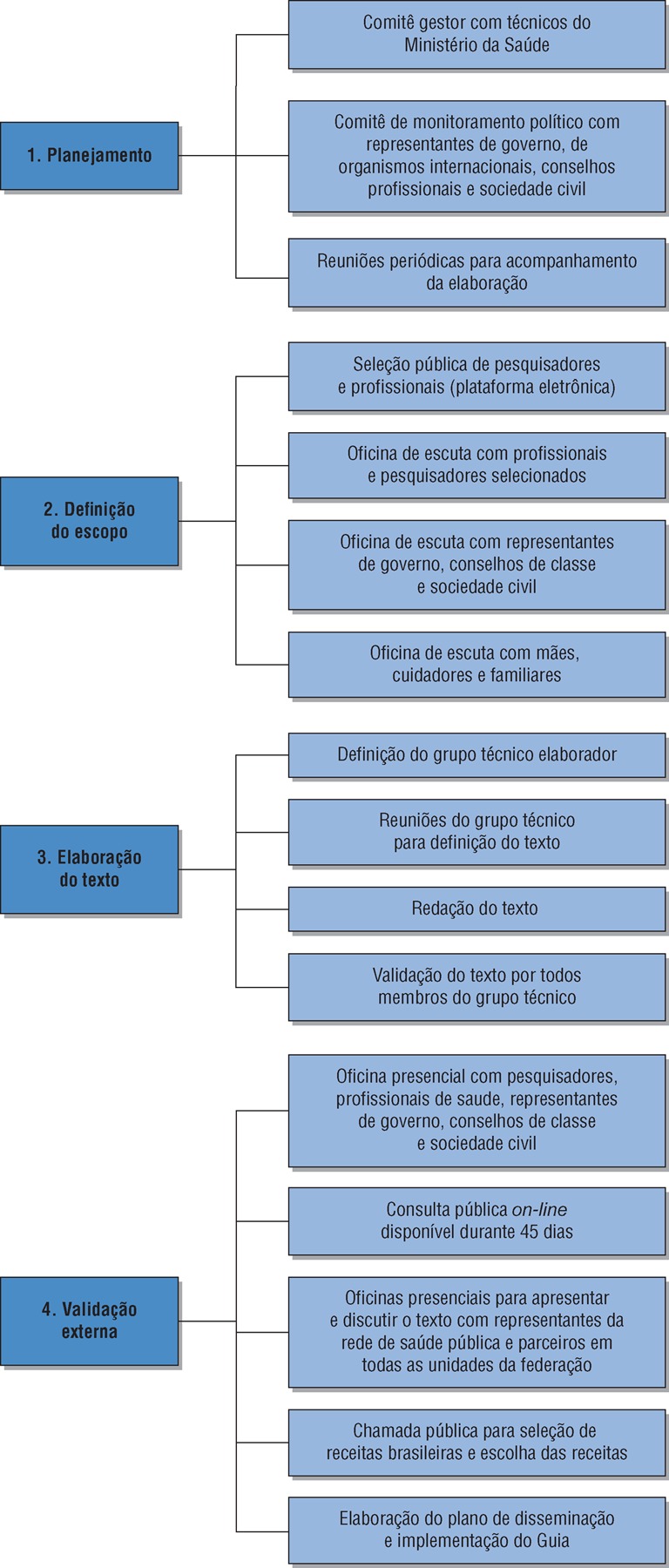
Fluxo de desenvolvimento de guias alimentares, Brasil

Para o desenvolvimento do Guia Alimentar para Crianças Brasileiras Menores de 2 Anos, foram acrescentadas, em relação ao desenvolvimento do Guia Alimentar de 2014, novas etapas de escuta envolvendo diferentes atores estratégicos (diversos setores do governo, pesquisadores, profissionais de saúde, representantes da sociedade civil, organismos internacionais, representantes de categorias de profissionais de saúde, mães, pais e cuidadores), bem como qualificação do processo de acompanhamento, monitoramento e elaboração do documento. Os passos do desenvolvimento estão descritos na figura 1.

Para a condução de todo o processo, foram definidos dois comitês: um comitê gestor, composto por técnicos do Ministério da Saúde, responsável pela definição do plano de elaboração do Guia com a definição das etapas e coordenação do processo de elaboração; e um comitê de monitoramento político, composto por representantes do governo (das áreas da saúde, educação, assistência social), de organismos internacionais, conselhos profissionais e da sociedade civil organizada. Foram realizadas reuniões periódicas para acompanhamento do processo de elaboração do documento. O grupo de monitoramento político se reunia duas vezes por ano.

A seguir, a definição do que deveria conter o Guia Alimentar envolveu quatro etapas, com três oficinas de escuta. Primeiramente, realizou-se uma seleção pública, por meio de plataforma eletrônica, de pesquisadores e profissionais de saúde atuantes ou com experiência na área para participação no processo. Os candidatos foram selecionados com base em critérios preestabelecidos pelo Comitê Gestor. A segunda etapa foi uma oficina de escuta na qual os pesquisadores e profissionais de saúde selecionados por meio de chamada pública discutiram o cenário alimentar e nutricional das crianças e definiram o escopo da nova edição do Guia. Na terceira etapa, realizou-se um oficina de escuta com atores-chave – representantes governamentais de diversos setores, pesquisadores, profissionais de saúde e educação, conselhos de classe e da sociedade civil. Finalmente, na quarta etapa, foram realizadas oficinas de escuta com mães, cuidadores e familiares de crianças que frequentavam ambulatórios e unidades básicas de saúde (UBS) para consultas de puericultura ou creches para bebês brasileiros de 6 a 24 meses, ou familiares ligados a movimentos sociais. Com base nas etapas de escuta, técnicos do Ministério da Saúde elaboraram um documento-síntese que continha um diagnóstico da situação alimentar e nutricional da população e uma proposta de escopo da abordagem para a nova edição do Guia.

Outra etapa importante foi a elaboração do texto do Guia Alimentar, que foi realizada pelo grupo técnico elaborador, composto por pesquisadores de diversas universidades e regiões e representantes do Ministério da Saúde. O grupo trabalhou durante 3 anos. O texto foi elaborado com base em evidências científicas, com transparência e ausência de conflitos de interesse. Todos os capítulos foram amplamente discutidos e validados por todos os participantes do grupo técnico. Uma vez formulada a primeira versão, o texto passou pelas seguintes etapas de validação externa: oficina presencial com atores estratégicos (pesquisadores, profissionais de saúde, representantes governamentais, representantes da sociedade civil e representantes de conselhos de classe), consulta pública* on-line *(texto disponibilizado durante 45 dias para que toda a população pudesse fazer contribuições ao texto final) e oficinas presenciais para apresentar e discutir o texto com representantes da rede de saúde pública e parceiros em todas as unidades da federação, para aprimoramento do texto e mobilização para participação na consulta pública. As sugestões relevantes foram incorporadas ao texto em cada etapa.

Duas seleções públicas adicionais foram realizadas para seleção de receitas, sendo uma para escolher o comitê julgador das receitas e outra para selecionar receitas saudáveis das diferentes regiões. As receitas serão testadas e disponibilizadas em outra publicação.

Finalmente, durante o processo de elaboração técnica, foi criado o plano de disseminação e implementação do Guia. Uma das apostas para disseminar o conteúdo é a elaboração de um aplicativo para as famílias de crianças menores de 2 anos.

## CONSIDERAÇÕES SOBRE O PAPEL DOS GUIAS ALIMENTARES

As evidências nacionais e internacionais sobre o aumento do consumo de alimentos ultraprocessados e seu impacto negativo na saúde das populações têm-se fortalecido nos últimos anos. Entre 2000 e 2013, o crescimento do consumo de ultraprocessados foi de 43,7% no mundo inteiro, chegando a 114,9% na Ásia e Pacífico, 73,3% na Europa Oriental e 48% na América Latina (2).

No Brasil, estudos com dados provenientes da Pesquisa de Orçamentos Familiares (POF) 2008-2009 mostraram que os alimentos ultraprocessados representavam 21,5% da ingestão calórica diária dos brasileiros, enquanto os alimentos processados representavam 9,0% (10). No quartil da população que mais consome ultraprocessados, essa categoria de produtos contribuiu com 39,4% da energia da dieta; esses indivíduos possuíam risco de obesidade 37% maior (11). O Estudo Longitudinal de Saúde do Adulto (ELSA-Brasil) mostrou que os ultraprocessados responderam por 22,7% do total da energia consumida e que os indivíduos do maior quartil de consumo, com mais de 29% de sua energia proveniente desses alimentos, tiveram 31% mais chance de sobrepeso e 41% de obesidade (9).

No Canadá, um estudo relatou que 45% das calorias ingeridas eram provenientes de ultraprocessados. Os indivíduos com maior consumo desses alimentos (75,95% da energia ingerida) tinham 32% mais chance de obesidade do que aqueles com menor consumo (7). Para a população norte-americana, os ultraprocessados correspondiam a 58% da energia consumida e 89% do açúcar adicionado na dieta, sendo que aqueles que mais consumiam esses alimentos apresentaram 53% mais chance de obesidade (6). Na Europa, análise em 19 países mostrou que um aumento de 1% na disponibilidade domiciliar de alimentos ultraprocessados resultou no aumento de 0,25% na prevalência de obesidade (12). Um estudo francês mostrou que os ultraprocessados correspondiam a 18,4% dos alimentos consumidos e 35,9% do total calórico consumido, estando positivamente associados com sobrepeso e obesidade (13).

Há evidências, também, do efeito do consumo de alimentos ultraprocessados no desenvolvimento de doenças cardiovasculares e de câncer. Dados da coorte francesa já mencionada mostraram que um aumento de 10% na proporção de ultraprocessados na alimentação associou-se a um aumento de 12% no risco de desenvolvimento de câncer em geral e de 11% especificamente para câncer de mama (5). Outro estudo mostrou que a redução de 75% do consumo de gordura saturada, gordura trans, sal e açúcar provenientes de ultraprocessados e ingredientes culinários poderia reduzir em até 29% a mortalidade por doenças cardiovasculares (14). Coorte realizada com 8 451 adultos de meia-idade que se formaram em universidades na Espanha mostrou que, do total de 1 939 casos de sobrepeso e obesidade registrados durante aproximadamente 8,9 anos, aqueles que apresentaram maior consumo de ultraprocessados tinham maior chance (26%) de desenvolver sobrepeso e obesidade do que aqueles com consumo menor (15).

No Brasil, os guias alimentares têm um papel estratégico na indução de políticas públicas de alimentação e nutrição e na garantia do direito humano à alimentação e à soberania alimentar. Ao adotarem uma abordagem qualitativa e orientada pelo grau de processamento dos alimentos, os guias induzem, de forma consciente, uma concepção que visa, acima de tudo, à garantia da saúde e nutrição da população brasileira e supera, consequentemente, a ideia reducionista de serem apenas uma fonte para ações de educação alimentar e nutricional.

Em consonância com essa filosofia, publicou-se, no âmbito do Ministério da Saúde, uma normativa que proíbe a comercialização e a utilização de recursos federais para compra ou oferta de alimentos ultraprocessados prontos para o consumo dentro do órgão e de entidades vinculadas; em tramitação, enviados para a Casa Civil, estão dois projetos de lei elaborados pelo Ministério da Saúde, com base no Guia Alimentar para a População Brasileira, que versam sobre a restrição da comercialização e da publicidade de alimentos ultraprocessados nas escolas públicas e privadas brasileiras e a restrição da publicidade de alimentos ultraprocessados para o público infantil. Além disso, o nível de processamento de alimentos tem sido contemplado em todas as discussões do Ministério da Saúde com a Agência Nacional de Vigilância Sanitária (ANVISA) quanto ao modelo de rotulagem nutricional frontal, que busca dar maior transparência ao conteúdo de açúcar, gordura e sódio dos alimentos, em grande parte ultraprocessados, comercializados no país.

Mais de 100 países já desenvolveram guias alimentares. No entanto, a maioria ainda apresenta recomendações baseadas em grupos de alimentos, sem considerar o grau de processamento. Para difundir a importância de debater o processamento de alimentos no campo das políticas públicas de alimentação e nutrição, o Brasil, por meio do Ministério da Saúde, com apoio da Organização Mundial da Saúde (OMS), Organização Pan-Americana da Saúde (OPAS) e Organização das Nações Unidas para Alimentação e Agricultura (FAO), propôs a criação de uma rede internacional de guias alimentares baseados no nível de processamento de alimentos. A rede é um espaço de intercâmbio de experiências e colaboração entre os países da Região das Américas. Tem como objetivo apoiar os países na elaboração, implementação, monitoramento e avaliação de guias alimentares que incorporem o nível de processamento para classificar os alimentos, fazer recomendações de alimentação saudável e fomentar a utilização dos guias como indutores de políticas públicas para promover uma alimentação saudável que valorize alimentos *in natura* e minimamente processados e diminua o consumo de alimentos ultraprocessados. Até o momento, os integrantes da rede são Brasil, Colômbia, Costa Rica, México, Equador, Argentina, Chile, Uruguai e Canadá. A rede é aberta para adesão e participação de outros países.

As etapas do processo de trabalho desenvolvidas na elaboração dos guias alimentares brasileiros respeitaram pressupostos que se tornaram pilares importantes no processo de construção das recomendações. Como resultado do aprendizado na construção dos Guias Alimentares, destacamos a necessidade de dar ampla transparência ao processo de construção das novas recomendações; realizar escuta abrangente para incorporar demandas dos atores envolvidos no processo de alimentação e nutrição de crianças; dispor das melhores evidências científicas para construção das recomendações mais adequadas ao público a que se destinam os guias; e considerar a exigência da ausência de conflito de interesses para os indivíduos envolvidos na elaboração do documento.

Por fim, destaca-se que trazer à luz o debate do nível de processamento de alimentos em documento tão relevante e norteador de políticas públicas, como um guia alimentar, demarca uma posição política e reafirma a importância do fortalecimento das instituições governamentais e da sociedade civil para que o interesse de saúde pública prevaleça nos espaços decisórios.

## Contribuição das autoras.

GAB, ALPM, AMCL, HOMM, OM, ICMD e MLO elaboraram o projeto, redigiram a primeira versão do artigo, levantaram e analisaram as informações apresentadas e redigiram a versão final do artigo. Todas as autoras revisaram e aprovaram a versão definitiva.

## Declaração.

As opiniões expressas no manuscrito são de responsabilidade exclusiva das autoras e não refletem necessariamente a opinião ou política da RPSP/PAJPH ou da Organização Pan-Americana da Saúde (OPAS).
